# Seasonal and Landscape‐Driven Variations in Forage Resources of *Apis mellifera scutellata* : Implications for Pollination Sustainability and Colony Health in Taita Taveta County, Kenya

**DOI:** 10.1002/ece3.71613

**Published:** 2025-06-27

**Authors:** Mary Chege, Mbatha B. Wambua, Wambua J. Kilonzo, Sevgan Subramanian, Beatrice T. Nganso

**Affiliations:** ^1^ International Centre of Insect Physiology and Ecology (Icipe) Nairobi Kenya

**Keywords:** DNA metabarcoding, exotic and native plants, honey bee nutrition, landscape composition, seasonality

## Abstract

Seasonality and land‐use change are key factors influencing forage availability for managed honey bee colonies, yet knowledge of forage identity and how these factors influence forage availability remains limited in Africa. To address these gaps, we used DNA metabarcoding to identify nectar and pollen plant species supporting the nutrition of the African savannah honey bee, *Apis mellifera scutellata* , across different land‐use/land cover types and seasons in Taita Taveta County, Kenya. We identified 224 forage plant species from 65 families, with Asteraceae, Fabaceae, and Myrtaceae being the top contributors. Forage availability was significantly influenced by landscape and season, with honey bees in agricultural lowland areas foraging on fewer and less diverse resources, particularly pollen, than those in forested highland and midland areas during the short dry season. Nectar plants (the primary source of carbohydrates that support energetic needs) were generally more diverse than pollen plants (the main source of proteins and lipids that support development and health). Exotic species dominated the nutrition of *A. m. scutellata* (67%) compared to native species (33%), raising concerns about the long‐term sustainability of local pollination networks, pollinator health, and productivity. Overall, these findings provide a foundation for future research on the comparative nutritional composition of both native and exotic plants across seasons, their impacts on honey bee health and productivity, and how the occurrence of exotic plants may affect local plant‐pollinator networks, to guide the development of nutrient‐rich forage landscapes for honey bees in this county.

## Graphical abstract

We used DNA metabarcoding to identify nectar and pollen plant species supporting honey bee nutrition across different landscapes and seasons in Taita Taveta County, Kenya. Forage availability was influenced by landscape and season, with exotic species (67%) dominating over native ones (33%), raising concerns about colony health and how the occurrence of exotic plants may affect local plant‐pollination networks. These findings highlight the need to understand the nutritional profiles of both native and exotic plants to develop balanced forage landscapes.

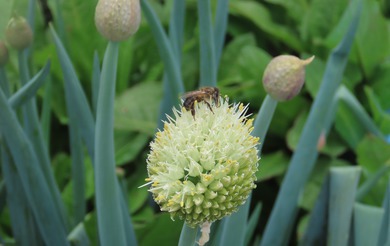

## 1 Introduction

Honey bees ( *Apis mellifera* L.) are the most widely managed pollinators globally (Potts et al. 2016). Their ability to form large colonies makes them valuable for boosting yields of approximately 75% of major agricultural crops that depend on animal pollination for human nutrition (Klein et al. 2007; Potts et al. 2010). In addition, they provide valuable hive products that support the livelihoods of millions of people (Nganso et al. 2024). To maintain these vital ecosystem services and ensure colony health, honey bees require access to diverse and nutrient‐rich floral resources within their surrounding landscapes (Brodschneider and Crailsheim 2010; Ansaloni et al. 2025).

Honey bees forage on plants primarily to collect nectar and pollen. Nectar, converted and stored as honey inside the hive, serves as their primary carbohydrate source, meeting the colony's energetic needs, while pollen supplies essential proteins, lipids, vitamins, secondary metabolites, and other nutrients crucial for larval development, cognitive function, and the overall colony fitness (Brodschneider and Crailsheim 2010). However, honey bee nutrition is complex because it is influenced by colony dynamics, spatiotemporal availability of food resources, food quality and diversity, seasonal changes, pest and pathogen pressure, agrochemical exposure, and beekeeping practices such as supplementary feeding (Haydak 1970; Brodschneider and Crailsheim 2010; Ghosh et al. 2020; Bryś et al. 2021; Tsuruda et al. 2021; Ansaloni et al. 2025). Since no single plant species can meet all their dietary needs due to spatiotemporal variations in their nutritional profiles, honey bees require access to a nutritionally rich and diverse array of plant species (Pernal and Currie 2001; Leonhardt and Blüthgen 2012; Di Pasquale et al. 2016; De Vere et al. 2017). In fact, previous studies have demonstrated that high‐quality and diverse resources significantly boost colony health, longevity, and resilience to both biotic and abiotic stressors (Alaux et al. 2010; Di Pasquale et al. 2013; Wang et al. 2014; Smart et al. 2019). However, habitat loss and deterioration have considerably reduced forage availability and quality, leaving honey bee populations increasingly vulnerable to secondary stressors such as pests/pathogens, pesticides, and climate change, among others (Goulson et al. 2015; Kuchling et al. 2018; Dicks et al. 2021). These challenges are particularly severe in Africa, where habitat loss due to agricultural and urban expansion together with climate variability and pesticide use continue to threaten pollinator health (Vaudo et al. 2012; Ochungo et al. 2021, 2022; Dicks et al. 2021).

Previous studies in Kenya (Ochungo et al. 2021, 2022) and South Africa (Vaudo et al. 2012) have demonstrated that landscape degradation negatively impacts pollen diversity, natural honey bee colony densities, and colony strength. A recent continental survey also reported that forage shortages caused by drought are a major factor contributing to increased managed honey bee colony losses in countries such as Kenya and Benin (Nganso et al. 2025). Despite these growing pressures, detailed knowledge of honey bee forage plants remains extremely limited in most parts of Africa. Identifying key nectar and pollen forage plants in different landscapes is critical for sustaining colony performance and their ecosystem services through targeted planting schemes, as has been implemented in the USA (Shuel 1992; Lowenstein et al. 2015; Vaudo et al. 2015; Dibble et al. 2020) and Europe (Comba et al. 1999; Baldock et al. 2015; UK Parliament 2017). Similar strategy could benefit African beekeeping, especially by integrating native plants to strengthen ecological resilience, given centuries of exotic plant introductions (Alpern 2008; Zhou et al. 2017; Van Wilgen et al. 2020).

In Africa, honey bee colonies rear brood year‐round, with peak swarming activity synchronized with flowering abundance (Hepburn and Radloff 1988). During forage scarcity, colonies must rely on available resources or abscond (depart their hives) in search of better habitats, as supplementary feeding is rarely practiced among African beekeepers (Nganso et al. 2025). A recent study in Eastern Kenya used melissopalynology to determine the botanical origin of one resource type (e.g., pollen) collected by *A. m. scutellata* between May 2017 and November 2018, and evaluate the impact of land use/land cover (LULC) types on pollen diversity (Ochungo et al. 2021). Given that nectar and pollen serve different nutritional functions within the colony, there is a need to gather knowledge of nectar and pollen forage plants across different Kenyan landscapes.

In the current study, we aimed to assess how LULC types and seasonality influenced the diversity and abundance of nectar and pollen forage plant species for *A. m. scutellata* colonies in Taita Taveta county, Kenya. We leveraged on recent advances in DNA metabarcoding, which provide a more efficient and high‐resolution taxonomic method for identifying both nectar and pollen forage plant species of honey bees (Do et al. 2022). This method has facilitated recent studies on pollinator ecology and plant‐pollinator interactions (Leponiemi et al. 2023; Tommasi et al. 2023; Balvino‐Olvera et al. 2024; Ndungu et al. 2024). Additionally, the native versus exotic status of the identified plant species and their reported occurrence in Kenya through open‐access biodiversity platforms were examined to help guide landscape enrichment schemes with appropriate floral resources for honey bees in the county.

## 2 Materials and Methods

### 2.1 Study Sites

The study was conducted in Taita Taveta county, located in the coastal region of Kenya. The county covers an area of approximately 17,084.1 km^2^ and has a population of about 340,671 people (KNBS 2019). Taita Taveta county was selected based on (i) its prominence in honey production within Kenya (ii) distinct LULC classes, and (iii) variations in weather and altitude (King et al. 2011; Abera et al. 2022). The county lies within the known distribution range of the African savannah honey bee, *A. m. scutellata* (Raina and Kimbu 2005). Its climate is influenced by South‐Easterly winds, resulting in bimodal rainfall pattern with two rainy seasons (March–May and October–December) and two dry seasons (January–February and June–September). Rainfall ranges from 157 to 341 mm in lower elevation areas to 265–1200 mm in the highlands, with an overall annual average of approximately 650 mm (Taita Taveta County Government 2013; Ogallo et al. 2019). Temperatures vary from 18.2°C in the highlands to 25°C in the lowlands, averaging 23°C annually (Taita Taveta County Government 2013). LULC analyses were conducted within a 3 km radius of each apiary or study site selected for sample collection to reflect the foraging range of honey bees (Figure S1) (Visscher and Seeley 1982). These analyses showed that lowland sites found in agricultural areas (< 1000 m a. s. l.) (e.g., Kipusi 3.46° S, 38.38° E, Mwakitala 3.52° S, 38.32° E, Mdaminyi 3.50° S, 38.33° E, Mazola 3.48° S, 38.41° E, and Ndighai 3.50° S, 38.33° E) are dominated by *Acacia‐Commiphora* bushlands, cultivated fields (mainly sisal and sorghum plantations), grassy patches, and wildlife conservation zones (Pellikka et al. 2009, 2018). During the 2023 sample collection period, the average annual temperature, humidity, and precipitation in the lowlands were 22.1°C, 74.4% and 87.1 mm, respectively. In contrast, tree‐dominated midland sites (1000–1500 m a. s. l.) (e.g., Chawia 3.48° S, 38.35° E, Mwawache 3.48° S, 38.36° E, and Ngeri 3.47° S, 38.35° E) and tree‐dominated highland sites (> 1500 m a.s.l.) (e.g., Kilera 3.43° S, 38.30° E, Maghimbinyi 3.37° S, 38.35° E, and Iyale 3.42° S, 38.35° E) are characterized by indigenous and planted forests, cropland patches, and agroforestry systems (Pellikka et al. 2009, 2018). In the midlands, the average annual temperature, humidity, and precipitation were 19.0°C, 77.0% and 105.6 mm respectively, while in the highland, they were 17.7°C, 77.2% and 103.9 mm, respectively.

### 2.2 Collection of Pollen and Honey Samples

At each apiary selected for sample collection, three queenright colonies, each housed in Langstroth hives containing 7–10 frames of bees, were randomly selected. Pollen was collected from returning foragers using traps installed at hive entrances and activated for five consecutive days monthly. To ensure all foragers passed through the traps, alternative hive entrances were sealed during this collection period (Figure S2). Collected pollen was transferred to sterile centrifuge tubes using sterilized forceps to prevent contamination. For nectar sampling, a 4 cm ×4 cm section of three randomly selected combs containing uncapped honey was sampled monthly from each colony. Uncapped honey was used as a proxy for nectar, as it reflects recently collected nectar from flowering plants actively foraged by bees. Comb sections were collected using sterile scalpels and stored in sterile containers. All samples were collected aseptically, with field personnel wearing gloves and using sterilized equipment for each hive to minimize contamination. All collected samples were immediately stored at −20°C to preserve DNA integrity and prevent microbial growth before further analysis. Sample collection was conducted during the short dry season (January–February), long rainy season (April–May), and long dry season (June–August) of 2023 to capture seasonal foraging patterns. Samples were collected during the third week of each month.

### 2.3 Sample Processing and DNA Extraction

For nectar samples, 10 mL of pooled nectar from each apiary per monthly collection was diluted in a 1:2 ratio using sterile double‐distilled water. The mixture was vortexed thoroughly, followed by centrifugation at 3234 *g* for 30 min using an Eppendorf 5804 R centrifuge. After discarding the supernatant, the pellet was rinsed with 10 mL of sterile double‐distilled water and centrifuged again at 3234 *g* for 15 min. The supernatant was discarded, and the pellet was resuspended in 1.5 mL of sterile double‐distilled water before transferring the sample into 2.0 mL microcentrifuge tubes and centrifuging at 3234 *g* for 30 min. The supernatant was discarded, and the pellet was used for DNA extraction. Also, 100 mg of pooled pollen samples from each apiary per monthly collection were processed by dissolving them in 1.5 mL of sterile double‐distilled water and centrifuged at 3234 *g* for 15 min. The supernatant was discarded, and DNA was extracted from the pollen pellet.

In total, 52 nectar and 52 pollen samples were processed for DNA extraction using the CTAB method, following the manufacturer's protocol. Briefly, 100 mg of each sample was homogenized in a Tissue lyser II (Qiagen) machine at 30 Hz for 10 min using 2 mm Zirconia beads (BioSpec) after adding 700 μL of CTAB solution (prepared by dissolving 20 g CTAB in 1 L of buffer: 100 mL 1 M Tris–HCL pH 8, 280 mL 5 M NaCl, 40 mL 0.5 EDTA pH 8, topped up to 1 L with double‐distilled water), 25 μL of proteinase K, and 2 μL β‐mercaptoethanol. The mixture was incubated at 55°C for 30 min, followed by an increase to 65°C for 2 h. The samples were then allowed to cool to room temperature for 20 min, with occasional inversion to ensure mixing. Next, 700 μL of chloroform: isoamyl alcohol (24:1) was added, and the samples were mixed by inversion for 5 min. After centrifugation at 3234 *g* for 10 min, the aqueous upper phase was transferred to a new microcentrifuge tube. To precipitate the DNA, 0.1 volume of 3 M sodium acetate (pH 5.2) and 2 volumes of ice‐cold absolute ethanol were added, followed by incubation at −20^0^ C overnight. Afterward, the samples were centrifuged at 3234 *g* for 10 min, and the supernatant was discarded. The DNA pellet was washed twice with 700 μL of 70% cold ethanol and centrifuged at 3234 *g* for 5 min. Thereafter, ethanol was discarded, and the tubes with DNA pellets were inverted on a clean laboratory paper towel under a laminar hood at room temperature to dry the pellet. The DNA was eluted in 70 μL of nuclease‐free water, and purity and quantity were assessed using a nanodrop spectrophotometer (Thermo Scientific Nano‐drop 2000 series).

### 2.4 DNA Sequencing and Bioinformatics Analysis

Genomic DNA from nectar and pollen samples was shipped to Macrogen in South Korea for amplification and sequencing of the ITS region using the Illumina MiSeq platform (Miseq V3‐300 PE, paired end). The ITS2 and ITS4 region was amplified using F5_ATGCGATACTTGGTGTGAAT and R5_TCCTCCGCTTATTGATATGC primers (Leponiemi et al. 2023; Rakotonirina et al. 2023; Ndungu et al. 2024). A total of 104 samples were shipped for sequencing, and 92 samples (40 nectar and 52 pollen) passed quality control checks. A total of 6,709,766 paired‐end raw sequences obtained across the 92 samples for ITS2 and ITS4 regions were analyzed using QIIME2 (version 2022.2.0) (Bolyen et al. 2019). These raw read sequences have been deposited in the National Center for Biotechnology Information (NCBI) under BioProject accession PRJNA872955. First, the paired end reads were imported into QIIME2, where a quality control check was performed. Primer sequences were trimmed using the Catadapt (version 3.5) tool integrated within QIIME2 environment with a maximum error of 0.2 for primers. The forward reads were truncated at 240 bp, whereas the reverse reads at 180 bp. Thereafter, the QIIME2 DADA 2 pipeline was used to trim low‐quality bases, merging the forward and reverse reads and removing any chimera and low‐quality reads using a quality score below 30. After filtering and denoising, 4,079,555 (60.80%) paired‐end reads were retained, all of which had more than 5000 reads. The sequences obtained were classified taxonomically to the species level using BLAST searches against the ITS database (ITS_eukaryote_sequences.tar.gz) on NCBI (https://ftp.ncbi.nlm.nih.gov/blast/db/). The *Taxonomizr* package in R‐software was used to complete the different taxonomic classification (Sherrill‐Mix 2017). After taxonomic classification, only taxa with more than 10 reads were retained. Species‐level identification in NCBI database was confirmed for sequences showing at least 97% identity. Species classification was also confirmed using the PLANTiTS database (Banchi et al. 2020).

### 2.5 Determining the Native Status of the Identified Plant Species and Their Occurrence Report in Open‐Access Biodiversity Platforms

The native or exotic status of the identified plant species was confirmed using Google, Plants of the World Online (https://powo.science.kew.org/), and iNaturalist (https://www.inaturalist.org/). In Google, the search phrase was used: Is “plant taxa” “native” in “Kenya?”. To determine whether the identified plant species had been previously reported in Kenya, two well‐known open‐access biodiversity databases, iNaturalist and GBIF (Global Biodiversity Information Facility) (https://www.gbif.org/), were used alongside Plants of the World Online.

### 2.6 Data Analysis

All analyses were conducted using R software (version 4.3.3) (R Core Team 2024). The read counts for plant species in nectar and pollen samples were first converted into percentages for normalization to account for technical artifacts before performing statistical analysis (De Vere et al. 2017; Leponiemi et al. 2023). To evaluate the impacts of LULC types (agricultural lowland areas, tree‐dominated midland and highland areas), seasons (long rainy season, short and dry seasons), sample types (nectar and pollen) and their interactions on plant species composition, a permutational multivariate analysis of variance (PERMANOVA) test with Bray–Curtis dissimilarity was run using the *vegan* package (Oksanen 2013). Shannon–Weaver diversity indices (H) for nectar and pollen plant species in each LULC type per sampling season were obtained using the *vegan* package. Thereafter, analyses of variance (ANOVA) followed by a post hoc Student–Newman–Keuls (SNK) test were conducted to compare species diversity among LULC types within each season. Before running ANOVA, the normality of the data and homogeneity of variances were confirmed using the Shapiro–Wilk test (*p* > 0.05) and Bartlett's test (*p* > 0.05), respectively. This statistical approach was used to compare bee forage species diversity across different seasons within each LULC type. To illustrate the differences observed, boxplots with error bars for mean diversity (H) were created using the *ggplot2* package (Wickham 2016). The *tidyverse* (Wickham et al. 2019) and *ggplot2* packages were used to generate stacked bar graphs showing the most abundant plant species providing nectar and pollen to the managed honey bee colonies. The *prop.test ()* function was used to compare the proportions of native and exotic bee forage plant species occurring in Kenya, as well as those reported in open‐source biodiversity databases. It is worth noting that pollen samples from the lowland during the long dry season were not included in these analyses due to unrepresentative sampling caused by limited floral resources and minimal bee foraging activity. This exclusion ensured more reliable comparisons across sites and seasons.

## 3 Results

### 3.1 Native and Exotic Bee Forage Plant Species Identified and Their Report in Open‐Access Biodiversity Platforms

**FIGURE 1:**
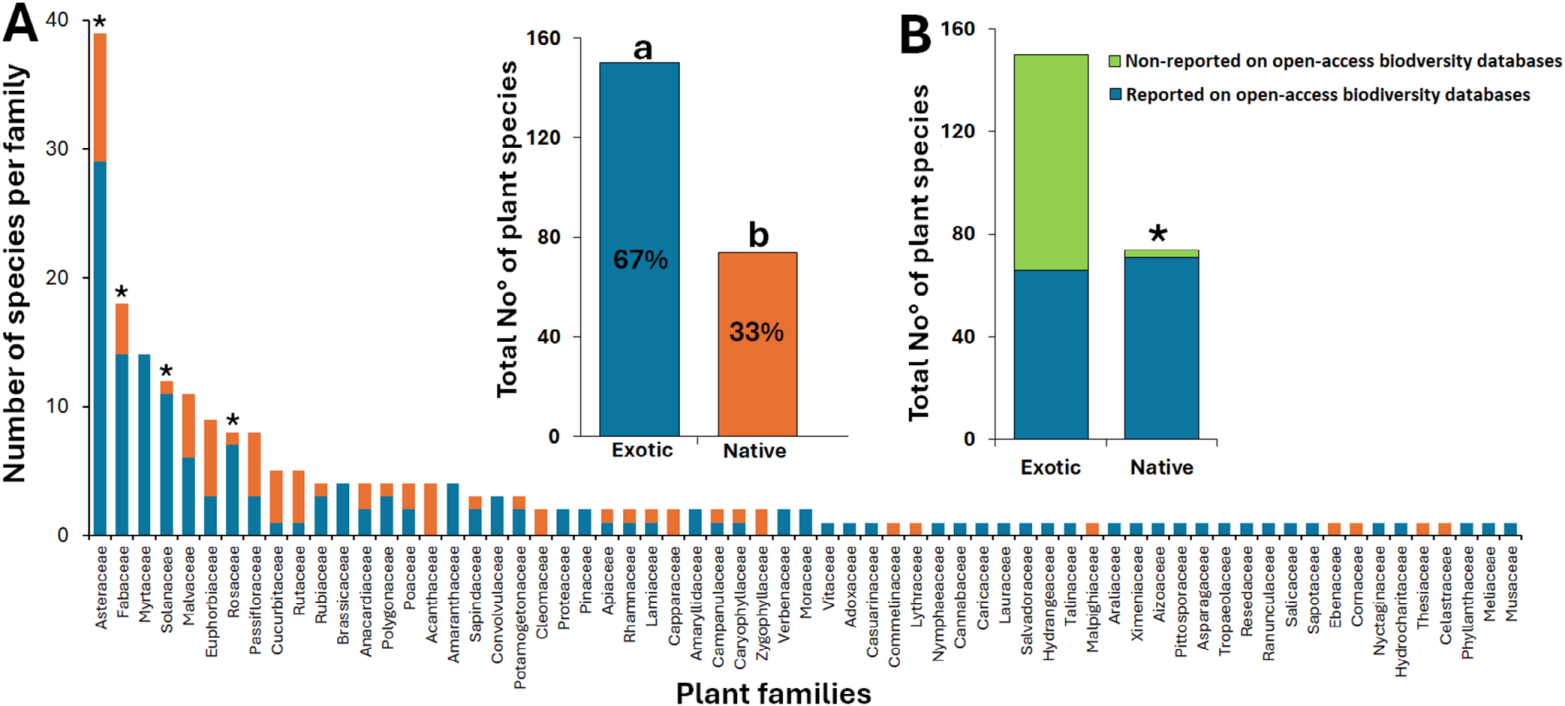
Proportion of native and exotic bee forage plants (A) and their occurrence in open‐access biodiversity databases (B). Bars with superscript letters (a and b) or asterisks (*) indicate significant differences in the proportion of native versus exotic plants, and in the proportion of those reported in open‐access databases versus those not reported, respectively (Chi‐square test, *p* < 0.05).

In this study, a total of 224 honey bee forage plant species from 65 families were identified (Table S1). A significant proportion of these plants were exotic in Kenya (67%), while only 33% were native (Chi‐Square = 50.22, df = 1, *p* < 0.001) (Figure 1A). The proportion of native versus exotic plant species differed significantly within certain families. Asteraceae (Chi‐Square = 4.39, df = 1, *p* < 0.05), Fabaceae (Chi‐Square = 9.0, df = 1, *p* < 0.01), Solanaceae (Chi‐Square = 13.50, df = 1, *p* < 0.001) and Rosaceae (Chi‐Square = 6.25, df = 1, *p* < 0.05) had a significantly higher proportion of exotic species compared to native ones (Figure 1A). These families were among the top 10 most abundant plant families providing forage resources for honey bees in the study sites. Additionally, the Myrtaceae family, also in the top 10, consisted entirely of exotic species. Interestingly, a significantly large proportion (94.5%) of the native bee forage plants were already reported in global biodiversity repositories as occurring in Kenya (Chi‐Square = 114.19, df = 1, *p* < 0.001) (Figure 1B).

### 3.2 Impact of Sample Type, LULC Composition, Seasonality, and Their Interaction on the Composition of Honey Bee Forage Resources

Our findings revealed that forage plant species composition was significantly influenced by sample type (nectar or pollen) (PERMANOVA; *Pseudo‐F* = 2.68; df = 1, *R* ^2^ = 0.02, *p* < 0.001), LULC composition (PERMANOVA; *Pseudo‐F* = 2.64; df = 2, *R* ^2^ = 0.04, *p* < 0.001), and season (PERMANOVA; *Pseudo‐F* = 5.42; df = 2, *R* ^2^ = 0.09, *p* < 0.001). Additionally, the interactions between sample type and season (PERMANOVA; *Pseudo‐F* = 2.94; df = 2, *R* ^2^ = 0.05, *p* < 0.001), sample type and LULC composition (PERMANOVA; *Pseudo‐F* = 1.74; df = 2, *R* ^2^ = 0.02, *p* < 0.01), and LULC composition and season (PERMANOVA; *Pseudo‐F* = 2.00; df = 4, *R* ^2^ = 0.06, *p* < 0.001) were all significant. The three‐way interaction between sample type, LULC composition, and season was also significant (PERMANOVA; *Pseudo‐F* = 1.50; df = 3, *R* ^2^ = 0.03, *p* < 0.01).

**FIGURE 2:**
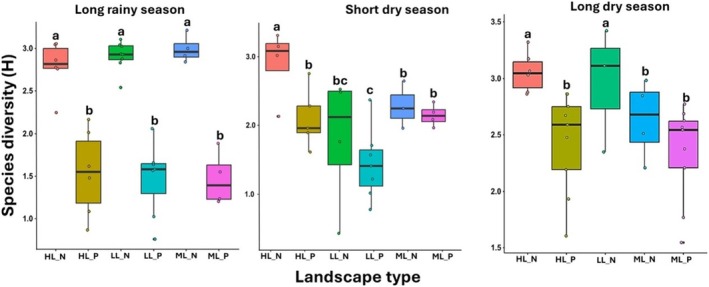
Comparison of the Shannon–Weaver diversity indices (H) for plant species among highland nectar (HL_N) and pollen (HL_P) samples, lowland nectar (LL_N) and pollen (LL_P) and midland nectar (ML‐N) and pollen (ML_P) during the long rainy season, as well as short and long dry seasons in Taita Taveta County, Kenya.

In fact, findings in Figure 2 showed that the diversity of nectar and pollen forage plant species significantly differed across LULC types during the long rainy season (ANOVA, *F*_(5,29)_ = 29.38; *p* < 0.001), short dry (ANOVA, *F*_(5,21)_ = 3.94; *p* < 0.05) and long dry seasons (ANOVA, *F*_(4,26)_ = 3.69; *p* < 0.05) (Figure 2). Specifically, nectar plant resources were consistently more diverse than pollen resources across all LULC types during the long rainy season. In the short dry season, honey plant species from the highland exhibited the highest diversity, while pollen plant species from the lowland had the lowest diversity. During the long dry season, nectar samples from the highland and lowland showed similar species diversity, which was significantly higher than that of pollen from the highland and both nectar and pollen from the midland. In general, we observed that nectar forage plants were more diverse than pollen forage resources across all LULC types regardless of the sampling season.

**FIGURE 3:**
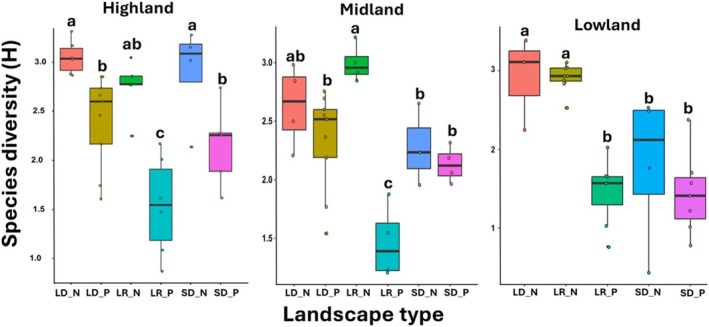
Comparison of the Shannon–Weaver diversity indices (H) for plant species among honey and pollen samples from the long dry season (LD_N, LD_P), long rainy season (LR_N, LR_P), and short dry season (SD_N, SD_P) within the highland, midland, and lowland regions.

Our findings further revealed a significant impact of seasonality on nectar and pollen bee forage plant diversity in the highland (ANOVA, *F*_(5,29)_ = 10.12; *p* < 0.001), midland (ANOVA, *F*_(5,22)_ = 9.35; *p* < 0.001) and lowland (ANOVA, *F*_(4,24)_ = 12.35; *p* < 0.001) (Figure 3). In both the highland and midland, bee pollen plant diversity was lowest during the long rainy season (LR_P), whereas it was comparable to nectar and pollen diversity from the short dry season (SD_H, SD_P). Across all landscapes, nectar samples generally exhibited higher species diversity than pollen samples. The highest species diversity was generally observed in nectar from the long dry (LD_H) and long rainy (LR_H) seasons, while the lowest diversity in pollen occurred in pollen from the long rainy season.

### 3.3 Seasonal Variation in Honey and Pollen Plant Resources Across LULC Types and Their Native Status

**FIGURE 4:**
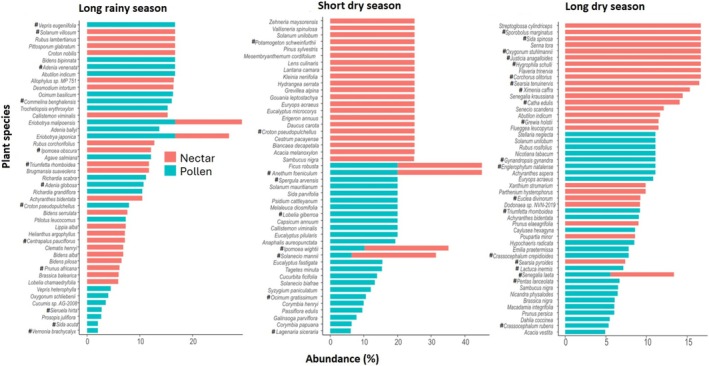
Nectar and pollen bee plant resources during the long rainy season, short and long dry seasons in the highland. The top 25 most abundant plant species across seasons were used. Only plant species with a mean proportion of at least 2% are shown. “**#**” indicates native plant species.

In this study, the diversity and contribution of nectar and pollen plant species varied across sampling seasons in the individual LULC type. Focusing on the top 25 most abundant nectar and pollen forage resources, certain plants exclusively provided nectar (honey sources) or pollen, while others served as dual resources across all landscapes. However, the extent to which plants contributed to both resources differed by landscape type. For example, a diverse range of plant species specialized in providing either nectar or pollen, while only few plants provided both nectar and pollen in the highland sites, (Figure 4). Native species from certain plant families also played a critical role in providing abundant forage resources to the honey bee colonies in this landscape (Figure 4). For example, Rutaceae (*Vepris eugeniifolia*), Solanaceae ( *Solanum villosum* ), Passifloraceae (*Adenia venenata*, *Adenia globosa*), Commelinaceae ( *Commelina benghalensis* ), Convolvulaceae ( *Ipomoea obscura* ), Malvaceae (*Triumfetta rhomboidei*, *Sida acuta* ), Euphorbiaceae ( *Croton pseudopulchellus* ), Asteraceae (*Centrapalus pauciflorus*, *Vernonia brachycalyx*), Rosaceae (*Prunus africa*), and Cleomaceae (*Sieruela hirta*) contributed to bee nutrition during the long rainy season. Other species, such as Potamogetonaceae (*Potamogeton schweinfurthii*), Euphorbiaceae ( *Croton pseudopulchellus* ), Apiaceae (*Anethum Foeniculum*), Caryophyllaceae ( *Spergula arvensis* ), Campanulaceae (*Lobelia giberroa*), Convolvulaceae (*Ipomoea wightii*), Asteraceae (*Solanecio mannii*), Lamiaceae ( *Ocimum gratissimum* ), Cucurbitaceae ( *Lagenaria siceraria* ), sustained substantially bee nutrition during the short dry season (Figure 4). During the long dry season, Poaceae (*Sporobolus marginatus*), Malvaceae ( *Sida spinosa* , *Corchorus olitorius* , *Grewia holstii*, *Triumfetta rhomboidea* ), Polygonaceae (*Oxygonum stuhlmannii*), Acanthaceae (*Justicia anagalloides*, *Hygrophila schulli*), Anacardiaceae (*Searsia tenuinervis*, *Searsia pyroides*), Ximeniaceae (*Ximenia caffra*), Celastraceae ( *Catha edulis* ), Cleomaceae ( *Gynandropsis gynandra* ), Sapotaceae (*Englerophytum natalense*), Ebenaceae (*Euclea divinorum*), Asteraceae ( *Crassocephalum crepidioides* , *Lactuca inermis* and *Crassocephalum rubens*), Fabaceae (*Senegalia laeta*), and Rubiaceae ( *Pentas lanceolata* ) provided essential nutritional support for honey bees (Figure 4).

**FIGURE 5:**
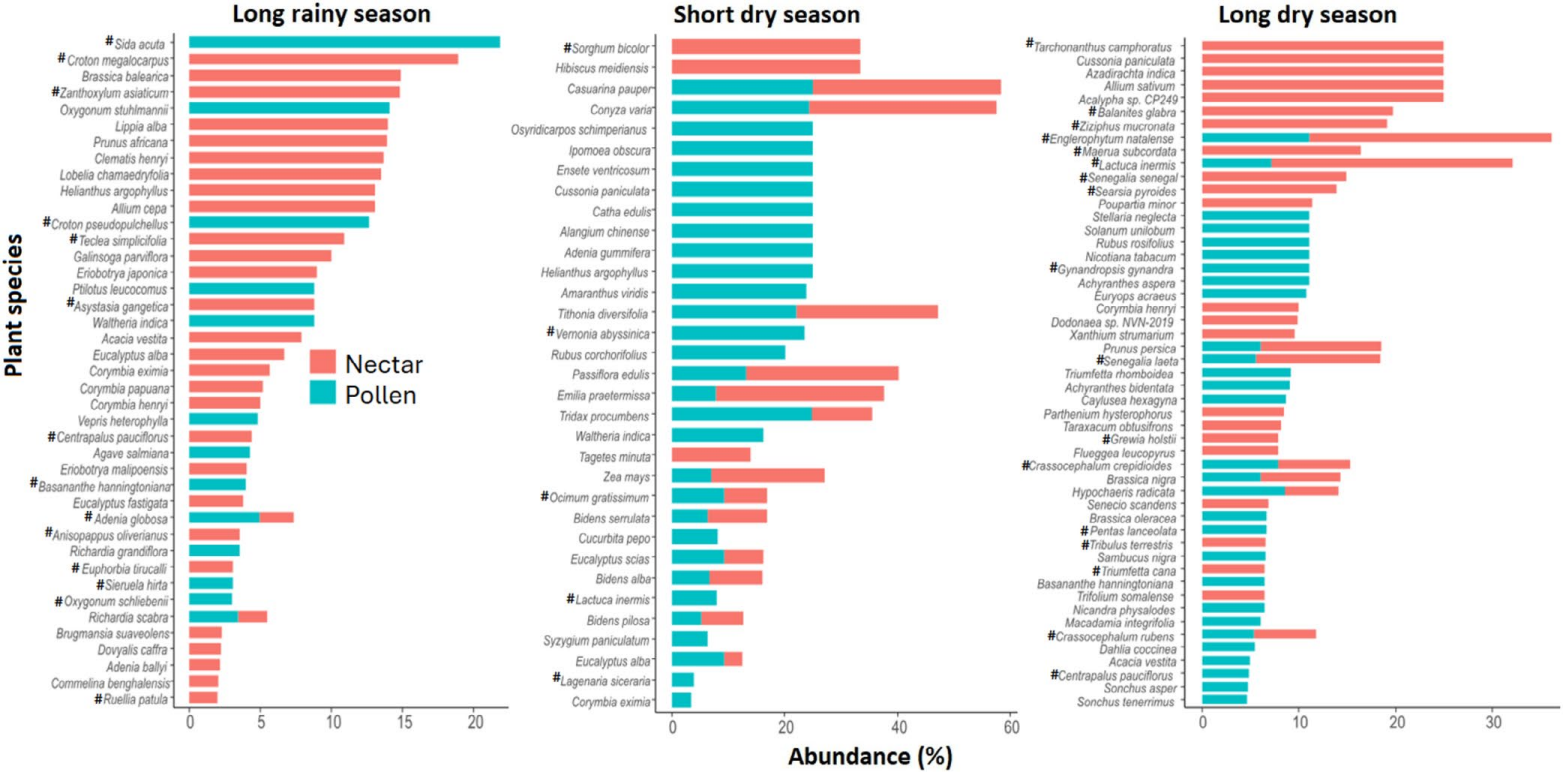
Nectar and pollen bee plant resources during the long rainy season, short and long dry seasons in the midland. The top 25 most abundant plant species across seasons were used. Only plant species with a mean proportion of at least 2% are shown. “**#**” indicates native plant species.

Unlike the highlands where more specialized separation between nectar and pollen sources were observed, the midland featured a considerable number of plant species serving as dual‐resource providers, especially during the short and long dry season (Figure 5). Key native bee forage plants in the midland during the long rainy season included species from Malvaceae ( *Sida acuta* ), Euphorbiaceae ( *Croton megalocarpus* ), Asteraceae (*Anisopappus oliverianus*), Cleomaceae (*Sieruela hirta*), Rutaceae (*Zanthoxylum asiaticum* and *Teclea simplicifolia*), Acanthaceae ( *Asystasia gangetica* and *Ruellia patula* ), Asteraceae (*Centrapalus pauciflorus*), Passifloraceae (*Basananthe hanningtoniana* and *Adenia globosa*), Polygonaceae (*Oxygonum schliebenii*) (Figure 5). During the short dry season, important native bee forage plants included species from Poaceae ( *Sorghum bicolor* ), Asteraceae (*Vernonia abyssinica* and *Lactuca inermis*), Lamiaceae ( *Ocimum gratissimum* ), Cucurbitaceae ( *Lagenaria siceraria* ) (Figure 5). However, during the long dry season, native bee forage plants comprised species from Asteraceae (*Tarchonanthus camphoratus* and *Lactuca inermis*), Zygophyllaceae (*Balanites glabra*), Rhamnaceae (*Ziziphus mucronate*), Sapotaceae (*Englerophytum natalense*), Capparaceae ( *Maerua subcordata* ), Fabaceae (*Senegalia Senegal* and *Senegalia laeta*), Anacardiaceae (*Searsia pyroides*), Cleomaceae ( *Gynandropsis gynandra* ), Malvaceae (*Grewia holstii* and *Triumfetta cana*), Asteraceae ( *Crassocephalum crepidioides* , *Crassocephalum rubens* and *Centrapalus pauciflorus*), Rubiaceae ( *Pentas lanceolata* ), and Zygophyllaceae ( *Tribulus terrestris* ) (Figure 5).

**FIGURE 6:**
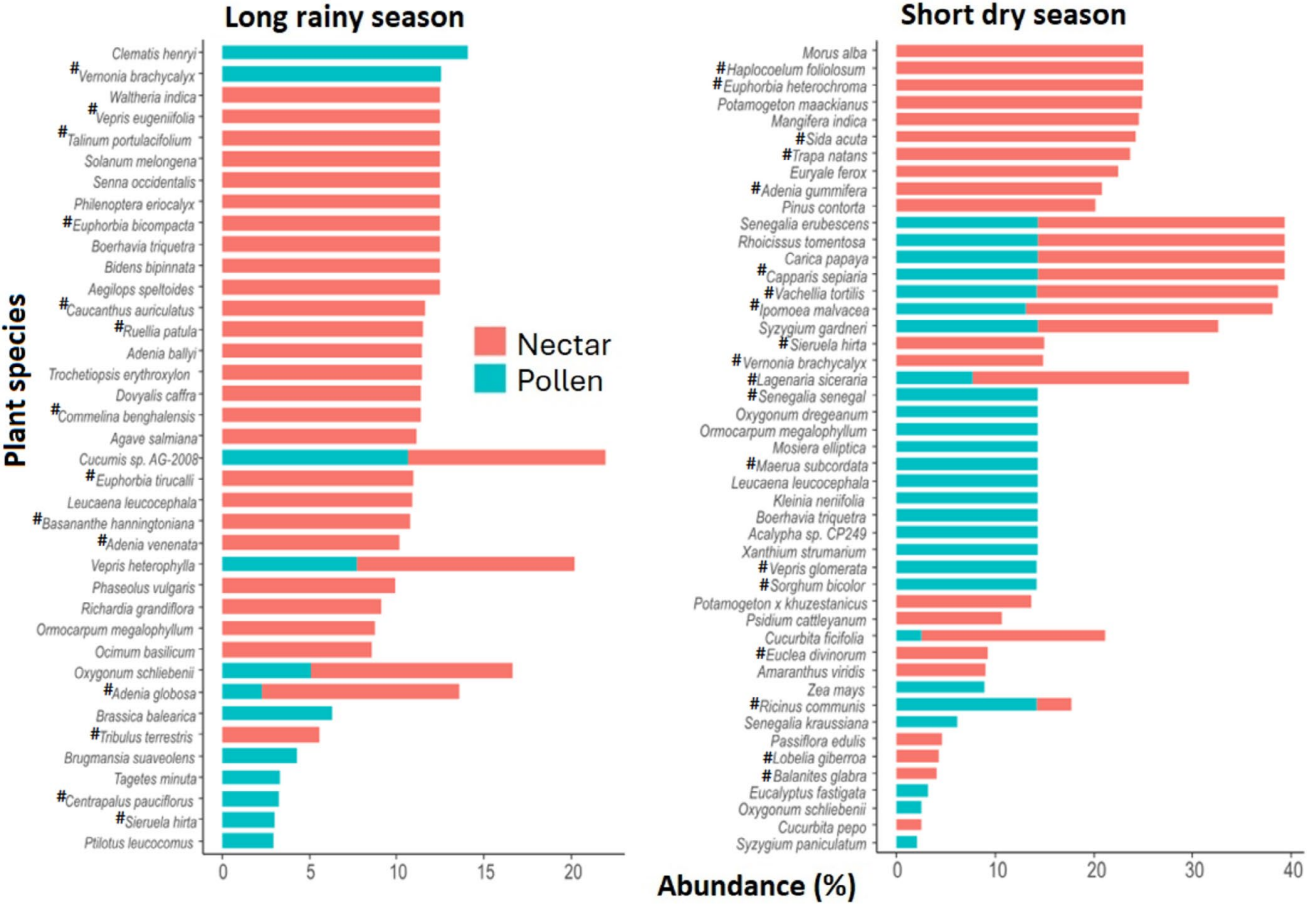
Nectar and pollen bee plant resources during the long rainy season, short and long dry seasons in the lowland. The top 25 most abundant plant species across seasons were used. Only plant species with a men proportion of at least 2% are shown. Are ranked by mean proportional abundance across. “**#**” indicates native plant species.

Like in the midlands, the lowland landscape featured species that provided both nectar and pollen during the short dry season, whereas the highest specialization of plant species offering either resource was observed during the long rainy season (Figure 6). During the long rainy season, prominent native plant families in this landscape included Asteraceae (*Vernonia brachycalyx* and *Centrapalus pauciflorus*), Rutaceae (*Vepris eugeniifolia*), Talinaceae ( *Talinum portulacifolium* ), Euphorbiaceae ( *Euphorbia bicompacta* and *Euphorbia tirucalli* ), Malpighiaceae (*Caucanthus auriculatus*), Acanthaceae ( *Ruellia patula* ), Commelinaceae ( *Commelina benghalensis* ), Passifloraceae (*Basananthe hanningtoniana*), Passifloraceae (*Adenia venenata* and *Adenia globosa*), Zygophyllaceae ( *Tribulus terrestris* ) and Cleomaceae (*Sieruela hirta*) (Figure 6). During the short dry season, dominant native plant families included Sapindaceae (*Haplocoelum foliolosum*), Euphorbiaceae ( *Euphorbia heterochroma* and *Ricinus communis* ), Malvaceae ( *Sida acuta* ), Lythraceae ( *Trapa natans* ), Passifloraceae (*Adenia gummifera*), Capparaceae (*Capparis sepiaria*, *Maerua subcordata* ), Fabaceae ( *Vachellia tortilis* and *Senegalia Senegal*), Convolvulaceae (*Ipomoea malvacea*), Cleomaceae (*Sieruela hirta*), Asteraceae (*Sieruela hirta*), Cucurbitaceae ( *Lagenaria siceraria* ), Rutaceae (*Vepris glomerata*), Ebenaceae (*Euclea divinorum*), Campanulaceae (*Lobelia giberroa*), and Caryophyllaceae (*Balanites glabra*) (Figure 6). Overall, most plant species identified herein specialized in providing either nectar or pollen rather than both, and this pattern was observed consistently across seasons in each landscape type (Figures S3–, S5).

Considering all the nectar and pollen plant species identified herein, the venn diagram illustration (Figure S6) supports the finding that the number of plant species contributing to nectar was substantially higher, reaching 53 species, while pollen‐providing species were as low as 5 species in some landscapes. An exception was observed in the midland landscape, where the number of pollen‐providing species (up to 40 species) exceeded nectar‐providing species.

## 4 Discussion

Our findings revealed the complex relationship between honey bee foraging patterns and environmental heterogeneity in Taita Taveta county, Kenya. Both LULC characteristics and seasonal patterns significantly influenced the availability of forage for honey bees. During the short dry season, the highland and midland study sites, which were dominated by tree cover, supported greater forage plant diversity, particularly for pollen, than the agricultural lowland sites (Figure 2). This difference is likely due to the harsher environmental conditions in the lowland areas, such as higher temperatures and the dominance of cultivated fields and grassy patches, which may limit the variety of flowering plants. Notably, Taita Taveta county is largely arid and semiarid (89%) (Rotich and Musyimi 2023), and yet the role of large plantations of drought‐resistant crops like sisal and sorghum in supporting honey bee nutrition, especially during the dry seasons, remains poorly understood in the lowlands. Previous studies have reported that dry climates have reduced honey bee forage plants by 50% (Ruto 2018) and caused 36%–70% colony losses in this county (Ruto 2018; Nganso et al. 2025), highlighting the negative impact of climate variability on honey bee health.

Our findings also showed that the most abundant nectar and pollen resources were generally distinct across seasons within the individual LULC type (Figures 4, 5, 6), suggesting potential nutritional stress during resource‐scarce periods, particularly in the agricultural lowland areas with low floral diversity. It was also intriguing to find that nectar plant diversity was generally higher than pollen diversity across all LULC types and seasons (Figure S3). This finding raised important ecological questions: Are honey bees prioritizing nectar over pollen collection to meet their carbohydrate needs? Does lower pollen diversity suggest nutritional efficiency or adaptation to the higher nectar‐to‐pollen diversity? How do plant traits and landscape features influence this pattern? Finally, what are the long‐term effects of this pattern on colony performance? Investigating these interesting questions will inform sustainable landscape management strategies for bee health in Taita.

It is worth noting that pollen in particular is the most significant source of honey bee nutrition, providing amino acids, lipids, secondary metabolites, minerals and vitamins essential for the proper functioning of the honey bee colonies (Brodschneider and Crailsheim 2010; Ansaloni et al. 2025). While a balanced intake of all these nutrients is necessary, lipid intake, especially the essential polyunsaturated fatty acids like linolenic acid (omega‐3) and linoleic acid (omega‐6) are often the most critical (Arien et al. 2020). These fatty acids are acquired solely via dietary pollen (Arien et al. 2020) and their deficiencies can impair honey bee learning and memory abilities, foraging efficiency, and other important developmental processes (Manning 2001; Arien et al. 2015, 2020), especially in disturbed habitats (Naug 2009; Scheper et al. 2014). These lipids also serve as an energy reserve during periods of low nectar and pollen availability in the landscape (Kunert and Crailsheim 1988; Manning 2001). However, the nutrient composition of both pollen and nectar vary widely among plant species due to species‐specific differences in the metabolic pathways and biochemical synthesis (Roulston et al. 2000; Nicolson 2011). Environmental factors such as soil quality, water availability, and climatic conditions (temperature and rainfall) also contribute to this variation (Wilson Rankin et al. 2020; Plos et al. 2023; Thuma et al. 2023). For instance, Ochungo et al. (2021) reported that in Kenya, seasonal variation has a stronger influence on pollen protein content than geographical differences. This variation underscores the need for promoting both nectar and pollen plant diversity across landscapes, particularly in the lowland areas where nutritional stress is more likely to occur, to ensure a year‐round balance diet for managed colonies in Taita Taveta county.

In this study, the top 10 plant families supporting the most honey bee nutrition (Asteraceae, Fabaceae, Myrtaceae, Solanaceae, Malvaceae, Euphorbiaceae, Rosaceae, Passifloraceae, Cucurbitaceae, and Rutaceae) were mostly exotic (Figure 1). Their dominance raised concerns about the long‐term stability of native bee forage plants and local pollination networks in Taita. While honey bees, as generalist foragers, can adapt to a wide range of floral resources including exotic species, native bees, often specialists, may respond differently to the loss of native plants depending on species‐specific traits, regions/continents, and/or landscape features (Kovács‐Hostyánszki et al. 2022; Zaninotto et al. 2023; Chitchak et al. 2024). Therefore, rather than prioritizing their removal, land managers should weigh the ecological risks and nutritional benefits of each species, aiming for a balanced approach that supports both biodiversity and pollinator health.

It is worth noting that the nutritional quality of floral resources from different families also varies. For example, large amounts of pollen produced by Asteraceae plants are generally low in protein content and digestibility, making them inadequate for sustaining bee nutrition alone (Vaudo et al. 2020; Bryś et al. 2021). Moreover, a previous study in South Africa showed that feeding managed honey bees with Asteraceae pollen (e.g., *Helianthus annuus* ) reduced ovarian development in worker bees compared to pollen from the Asphodelaceae family (e.g., *Aloe greatheadii* ) (Human et al. 2007). The fact that honey bees in the highland, midland, and lowland areas foraged on a diverse range of plant species across seasons suggests a possible adaptation to the low nutritional quality of Asteraceae‐dominated landscapes, an area which requires future investigation. In contrast, plants from the Fabaceae, Rosaceae, and Solanaceae families produce pollen with high protein‐to‐lipid (P:L) ratios (Vaudo et al. 2020) and a high lipid omega‐6:3 ratio (Arien et al. 2015). However, nectar‐rewarding families such as Malvaceae and Lamiaceae provide pollen with low P:L ratios (Vaudo et al. 2020). Plants from the Myrtaceae family, mostly represented by *Eucalyptus* spp. in this study, are good nectar sources (Table S1). However, *Eucalyptus* plants produce pollen poor in omega‐3 fatty acids and high in the omega‐6:3 ratio (Arien et al. 2015), which can impair honey bee learning ability and other development processes (Arien et al. 2015, 2020). This underscores the importance of not only providing sufficient amounts of these essential fatty acids but also maintaining a balanced omega‐6:3 ratio, as previously suggested by Arien et al. (2020). Other nectar‐rewarding families such as Acanthaceae, Rubiaceae, and Euphorbiaceae were identified in this study and previous reports in Kenya, including Taita (Onyango et al. 2019; Ruto 2018), but their nutritional profiles remain poorly understood. The limited understanding of bee nutritional ecology in Kenya and other parts of Africa (Nganso et al. 2024) highlights the need for more detailed research on forage quality.

## 5 Conclusions

This study found that *A. m. scutellata* in Taita Taveta county, Kenya generally sourced nectar and pollen from different plants, with forage availability significantly influenced by both seasonality and LULC composition. The dominance of exotic forage species among the identified forage plants calls for attention regarding the sustainability of local pollination networks, pollinator health, and productivity. To enhance bee health and ecosystem resilience, native forage plants must be promoted. However, since exotic plants are already present in the local ecosystems and contribute to honey bee nutrition as discussed above, future studies should compare native and exotic plants in terms of seasonal nutritional value, their impacts on honey bee health and productivity, as well as their influence on local plant‐pollinator networks. These insights will guide the design of forage‐rich landscapes that support healthy honey bee colonies and native pollinator species throughout the year.

## Author Contributions

**Mary Chege:** data curation (lead), formal analysis (lead), investigation (equal), writing – original draft (lead). **Mbatha B. Wambua:** investigation (lead), writing – original draft (equal). **Wambua J. Kilonzo:** investigation (equal), writing – original draft (equal). **Sevgan Subramanian:** conceptualization (equal), project administration (equal), writing – review and editing (equal). **Beatrice T. Nganso:** conceptualization (lead), data curation (lead), formal analysis (lead), methodology (lead), supervision (lead), validation (lead), visualization (lead), writing – original draft (lead), writing – review and editing (lead).

## Conflicts of Interest

The authors declare no conflicts of interest.

## Supporting information


**Figure S1.** Land use/land cover (LULC) map of Taita Taveta County for the year 2023, showing the selected study sites found within the cropland and tree cover areas.

**Figure S2.** Pollen trap fitted on a Langstroth hive for in‐hive pollen collection.

**Figure S3.** Nectar and pollen bee plant resources across seasons in the highland. The top 25 most abundant plant species across season were used. Only plant species with a mean proportion of at least 2% are shown and are ranked by mean proportional abundance across. “**#**” indicates native plant species.

**Figure S4.** Nectar and pollen bee plant resources across seasons in the midland. The top 25 most abundant plant species across season were used. Only plant species with a men proportion of at least 2% are shown. Are ranked by mean proportional abundance across. “**#**” indicates native plant species.

**Figure S5.** Nectar and pollen bee plant resources across seasons in the lowland. The top 25 most abundant plant species across season were used. Only plant species with a men proportion of at least 2% are shown. Are ranked by mean proportional abundance across. “**#**” indicates native plant species.

**Figure S6.** Number of shared and unique plant genera found in samples of pollen and honey during the long rainy season, short and long dry seasons in the highland, midland and lowland.

**Table S1.** Native and exotic status of 224 honey bee forage plants identified in Taita Taveta County, Kenya, and their occurrence in GBIF and iNaturalist databases.

## Data Availability

The data and R codes that support the findings of this study are available in the Dryad Digital repository (https://doi.org/10.5061/dryad.573n5tbkg).
