# Supplementary figures and images for: Seasonal and Landscape‐Driven Variations in Forage Resources of Apis mellifera scutellata : Implications for Pollination Sustainability and Colony Health in Taita Taveta County, Kenya
Source: Ecol Evol. 2025 Jun 27;15(7):e71613. doi: 10.1002/ece3.71613 (PMC12202974; doi:10.1002/ece3.71613)

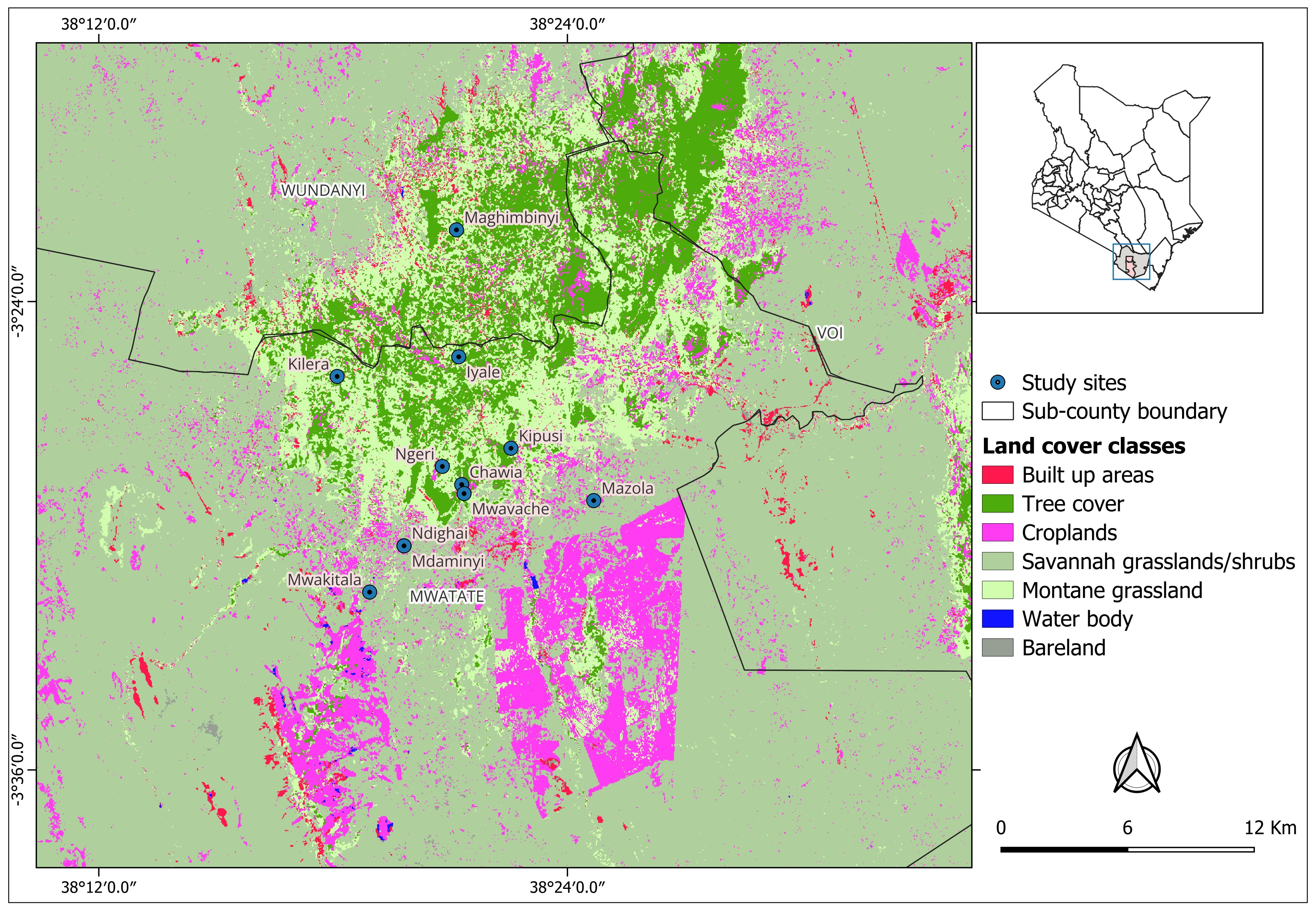

Supplement: Supplementary file 1 — Figure S1. Land use/land cover (LULC) map of Taita Taveta County for the year 2023, showing the selected study sites found within the cropland and tree cover areas. [file ECE3-15-e71613-s005.png]

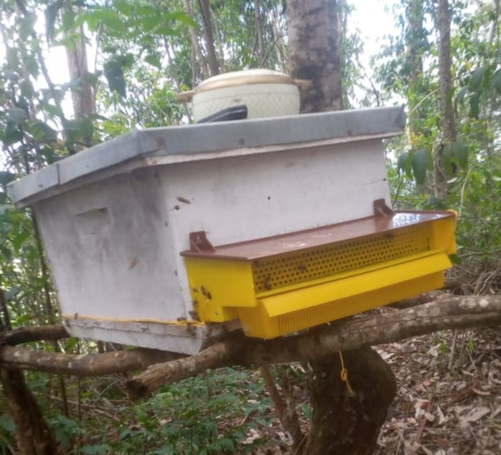

Supplement: Supplementary file 2 — Figure S2. Pollen trap fitted on a Langstroth hive for in‐hive pollen collection. [file ECE3-15-e71613-s004.png]

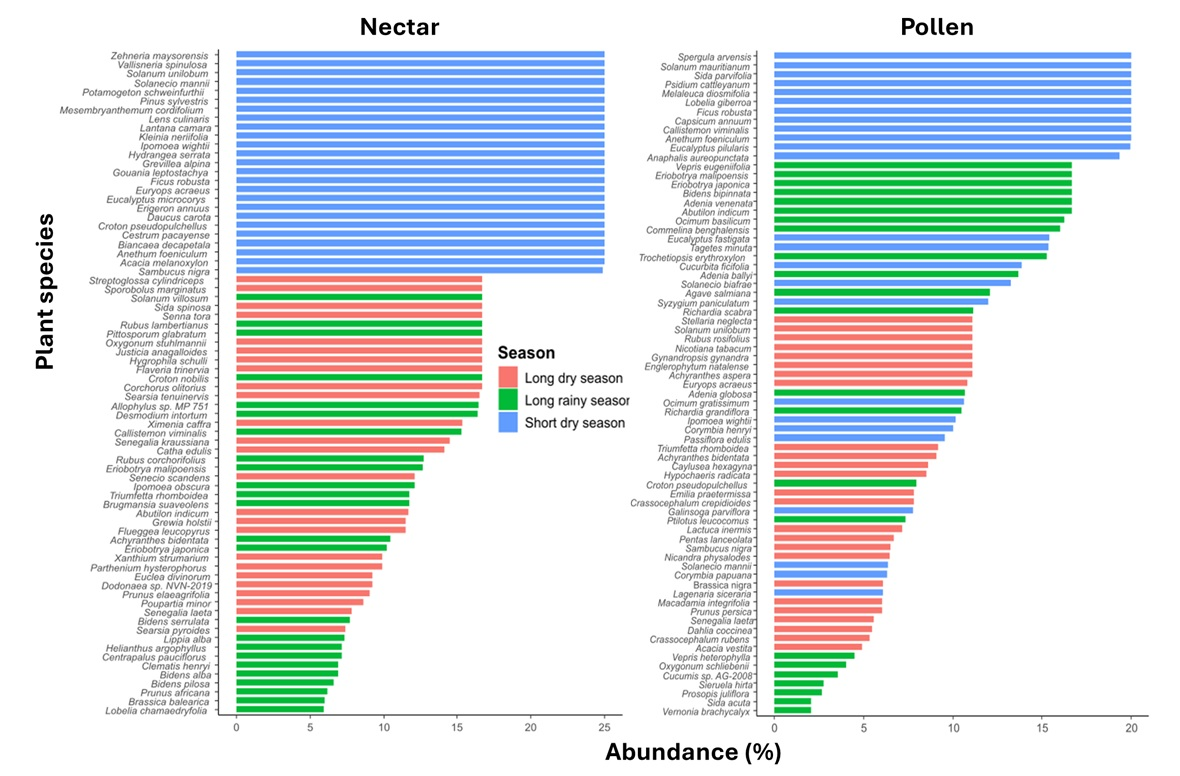

Supplement: Supplementary file 3 — Figure S3. Nectar and pollen bee plant resources across seasons in the highland. The top 25 most abundant plant species across season were used. Only plant species with a mean proportion of at least 2% are shown and are ranked by mean proportional abundance across. “#” indicates native plant species. [file ECE3-15-e71613-s007.tif]

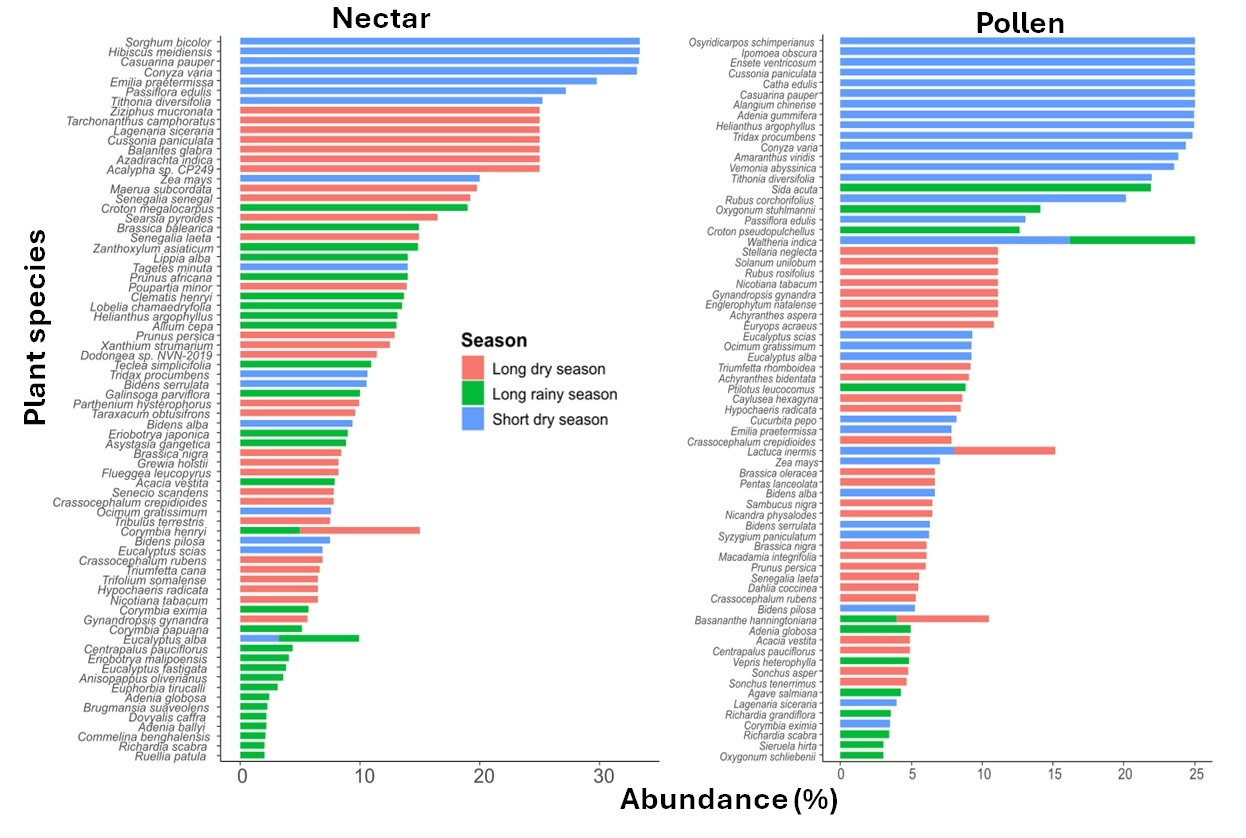

Supplement: Supplementary file 4 — Figure S4. Nectar and pollen bee plant resources across seasons in the midland. The top 25 most abundant plant species across season were used. Only plant species with a men proportion of at least 2% are shown. Are ranked by mean proportional abundance across. “#” indicates native plant species. [file ECE3-15-e71613-s003.tif]

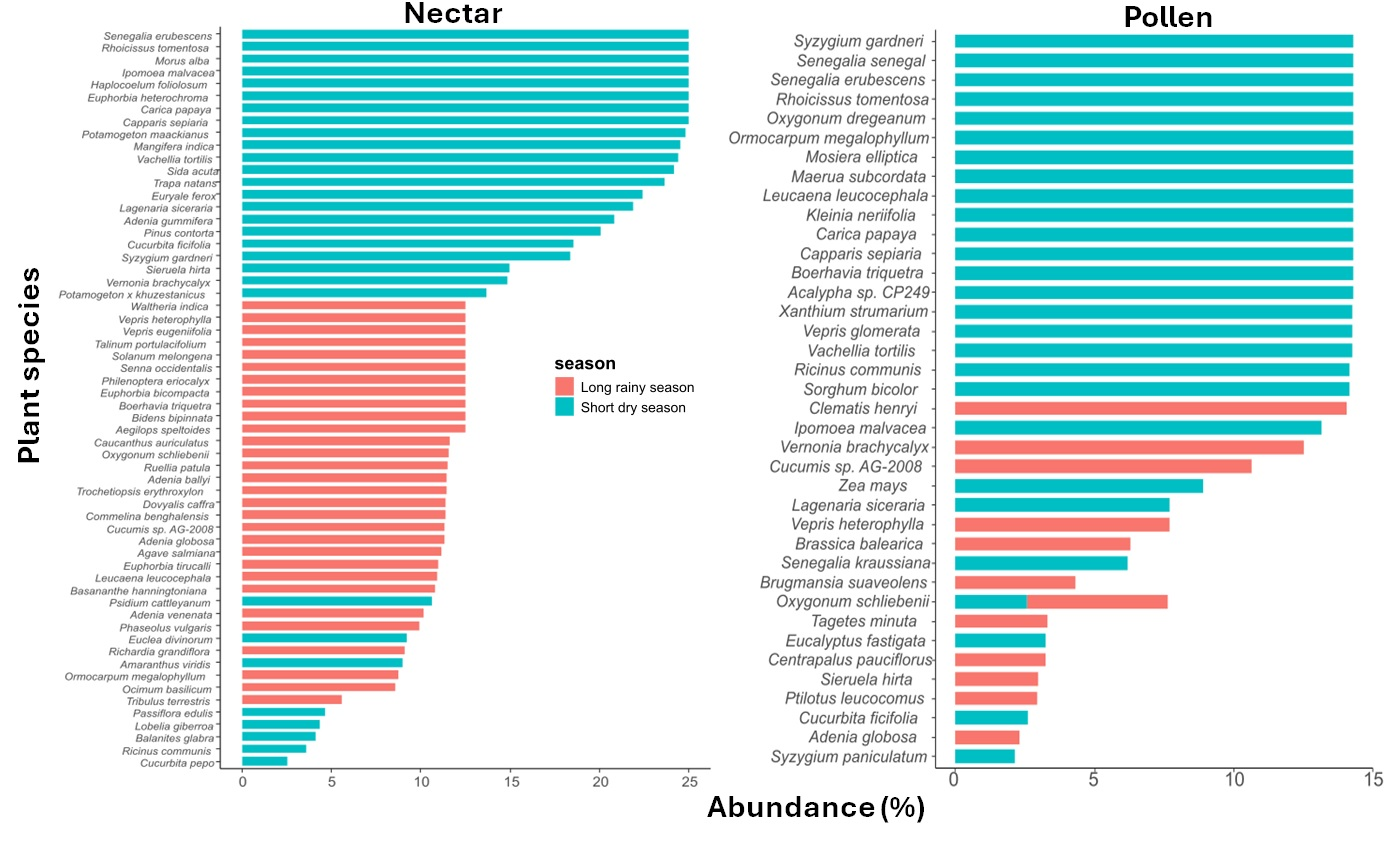

Supplement: Supplementary file 5 — Figure S5. Nectar and pollen bee plant resources across seasons in the lowland. The top 25 most abundant plant species across season were used. Only plant species with a men proportion of at least 2% are shown. Are ranked by mean proportional abundance across. “#” indicates native plant species. [file ECE3-15-e71613-s001.tif]

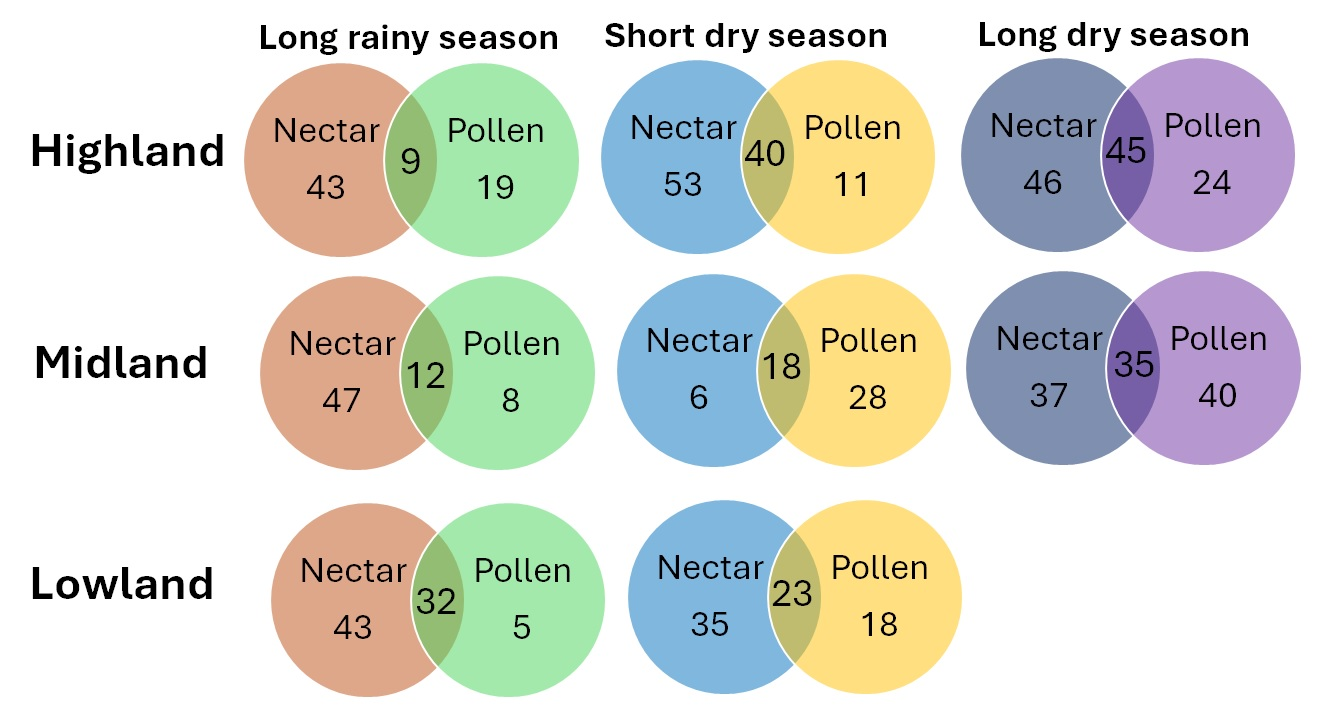

Supplement: Supplementary file 6 — Figure S6. Number of shared and unique plant genera found in samples of pollen and honey during the long rainy season, short and long dry seasons in the highland, midland and lowland. [file ECE3-15-e71613-s006.tif]
